# Covered Stenting for a Large Coronary Artery Aneurysm With Adjacent Stenosis in a Poor Surgical Candidate

**DOI:** 10.7759/cureus.28037

**Published:** 2022-08-15

**Authors:** Ghulam Mujtaba Ghumman, Muhammad Ahsan, Jay Shah, Kritika Luthra, Syed S Ali

**Affiliations:** 1 Internal Medicine, St. Vincent Mercy Medical Center, Toledo, USA; 2 Cardiology, Hartford Hospital, Hartford, USA; 3 Cardiology, St. Vincent Mercy Medical Center, Toledo, USA; 4 Interventional Cardiology, St. Vincent Mercy Medical Center, Toledo, USA

**Keywords:** guide catheter, covered stent, coronary angiography, coronary artery disease, size, aneurysm

## Abstract

Coronary artery aneurysms (CAAs) are being increasingly diagnosed with the advent of coronary angiography, and their management depends on the clinical presentation, size, and etiology of the aneurysm. Small aneurysms are usually managed with covered stents, while surgical intervention is considered for large aneurysms. We present a challenging case of a large CAA with adjacent coronary artery stenosis managed with guide extension catheter-assisted covered stent deployment as the patient was not a good surgical candidate.

## Introduction

Coronary artery aneurysms (CAAs) are defined as focal dilatation of a coronary artery segment that is 1.5-fold the adjacent normal diameter [1]. The incidence of coronary aneurysms is rising with the advent of coronary angiography and can range from 0.3 to 5.3% [2]. CAAs can be classified based on vessel wall structure, morphology, and size. True aneurysms (saccular or fusiform shaped) involve all three layers of the coronary arteries, while pseudoaneurysm only involves a single or double layer. The transverse diameter is greater than longitudinal in the saccular aneurysm and vice versa in the fusiform aneurysm [3]. The size of aneurysms varies, and they can be as large as >20 mm (or more than four times the diameter of the reference vessel), in which case they are considered giant aneurysms [4]. Atherosclerotic coronary artery disease (ACAD) is the most common cause of CAAs, with other less common causes including percutaneous coronary artery intervention, genetic predisposition especially in congenital coronary artery aneurysms, vasculitis or connective tissue disorders like Kawasaki disease, and Marfan syndrome [5]. We present a challenging case of a large CAA managed with a covered stent in a poor surgical candidate.

## Case presentation

**Figure 1:**
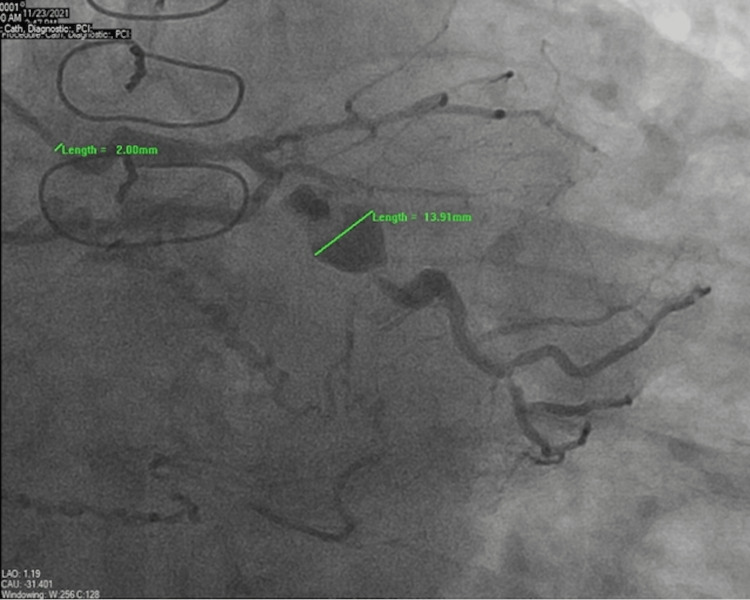
Coronary angiography shows a large fusiform aneurysm of the left circumflex artery (13.91 mm) with adjacent proximal and mid stenosis. The catheter diameter (2 mm) is shown in comparison to the aneurysm size

A 72-year-old male was brought to the hospital by emergency medical services for evaluation of chest pain. The patient had a past medical history of coronary artery bypass graft surgery (CABG) for severe multivessel coronary artery disease (CAD), essential hypertension, hyperlipidemia, and Type 2 diabetes mellitus. He reported sudden-onset sharp substernal chest pain, 8/10 in intensity, associated with nausea, diaphoresis, and dyspnea and relieved with 0.4 mg tablet of sublingual nitroglycerin. The patient did endorse episodes of similar but mild chest pain over the previous few weeks, especially on exertion. Vitals signs showed blood pressure 159/100 mmHg, pulse 86 beats per minute, and respiratory rate of 18 breaths per minute. His physical examination was unremarkable. An electrocardiogram (ECG) showed normal sinus rhythm with occasional premature ventricular complexes and nonspecific ST and T-wave changes. High sensitivity troponins were normal at 12 ng/L with no significant trend on repeat. The transthoracic echocardiogram (TTE) showed an ejection fraction of 55-60% without any obvious wall motion abnormality. Due to typical symptoms in the setting of known CAD, coronary angiography was performed, which showed 60-70% stenosis of the left main coronary artery (LMCA), and 100% stenosis of the proximal left anterior descending (LAD) artery. Left circumflex (LCx) had 90% proximal, 80-90% mid stenosis with a large fusiform (true) aneurysm (~14 mm), and distal obtuse marginal (OM) had 90% stenosis (Figure 1).

**Figure 2:**
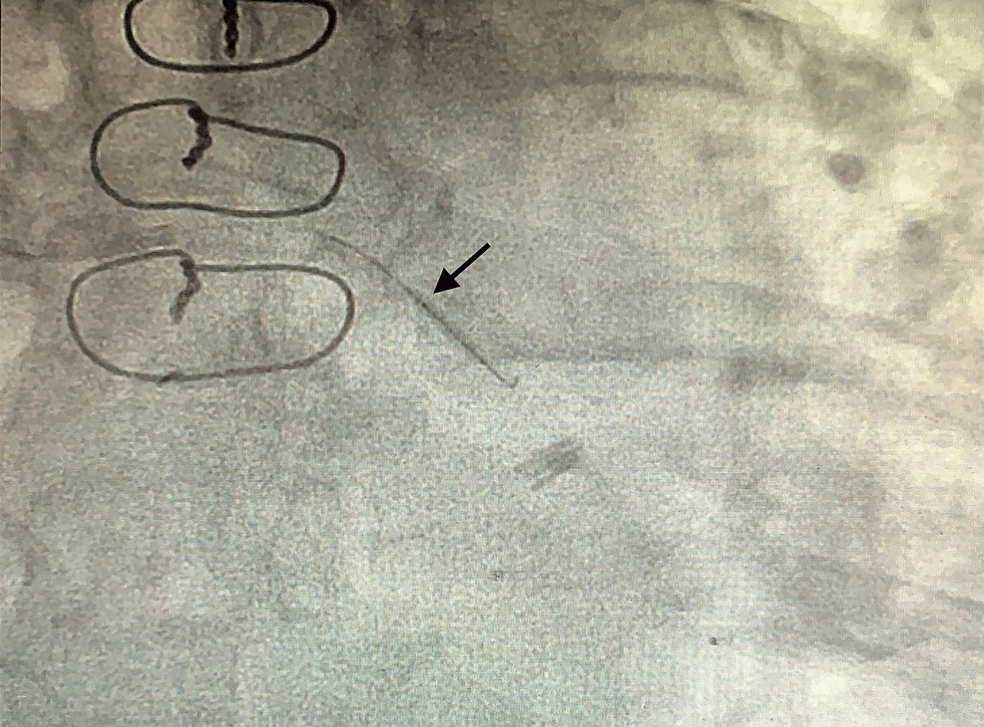
Coronary angiography showing guidewire with an angioplasty balloon catheter in the region of the aneurysm (black arrow)

**Figure 3:**
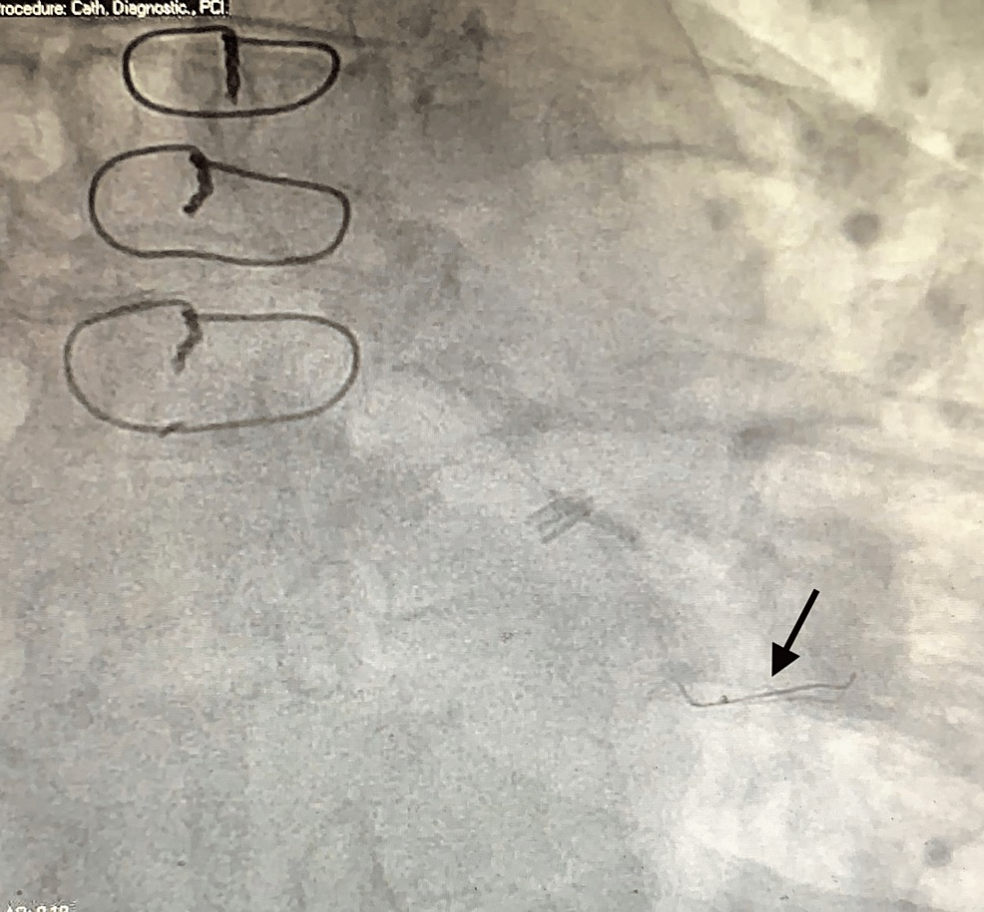
Coronary angiography showing that the guidewire is now parked more distally (black arrow)

The right coronary artery (RCA) was completely occluded but had good bridging collaterals from the left to supply the distal myocardium. Grafts engagement showed that the left internal mammary artery graft (LIMA) to LAD was evident with excellent collaterals to RCA, while saphenous vein grafts (SVG) to RCA and OM were occluded. The patient required a drug-eluting stent (DES) for LMCA, proximal LCx, and OM stenosis. Due to the risk of rupture, it was then decided to proceed with placing a covered stent for the mid LCx aneurysm and adjacent stenosis. The wiring of the aneurysmal segment stenosis was challenging as the guidewire was floating in the aneurysm area. The angioplasty balloon catheter helped the wire cross the distal end of the aneurysm (Figure 2,3).

**Figure 4:**
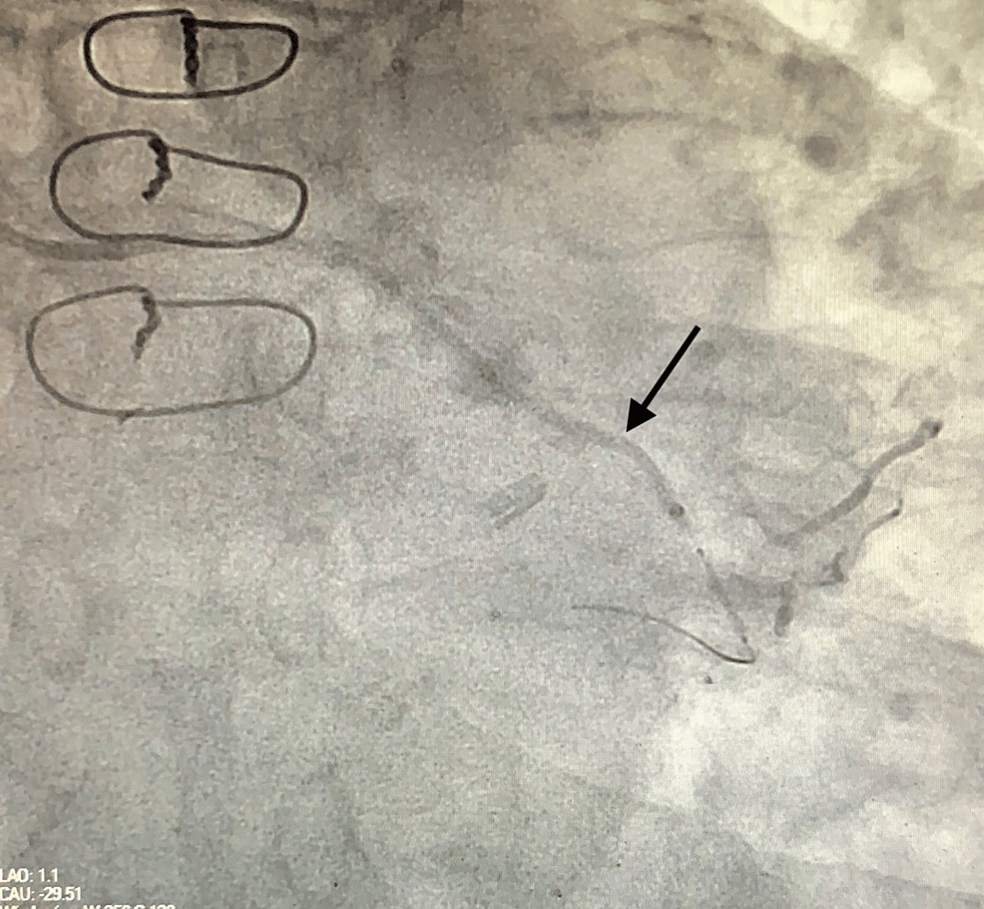
Coronary angiography showing guide extension catheter (arrow) used for the smooth delivery of the covered stent

After the balloon angioplasty of the mid LCx lesion, the initial attempt to deploy the covered stent using a guide catheter over the guidewire was not successful. A 6-Fr guide extension catheter (GuideLiner) was then inserted over the same guidewire, and it was advanced distal to the guide catheter (Figure 4).

**Figure 5:**
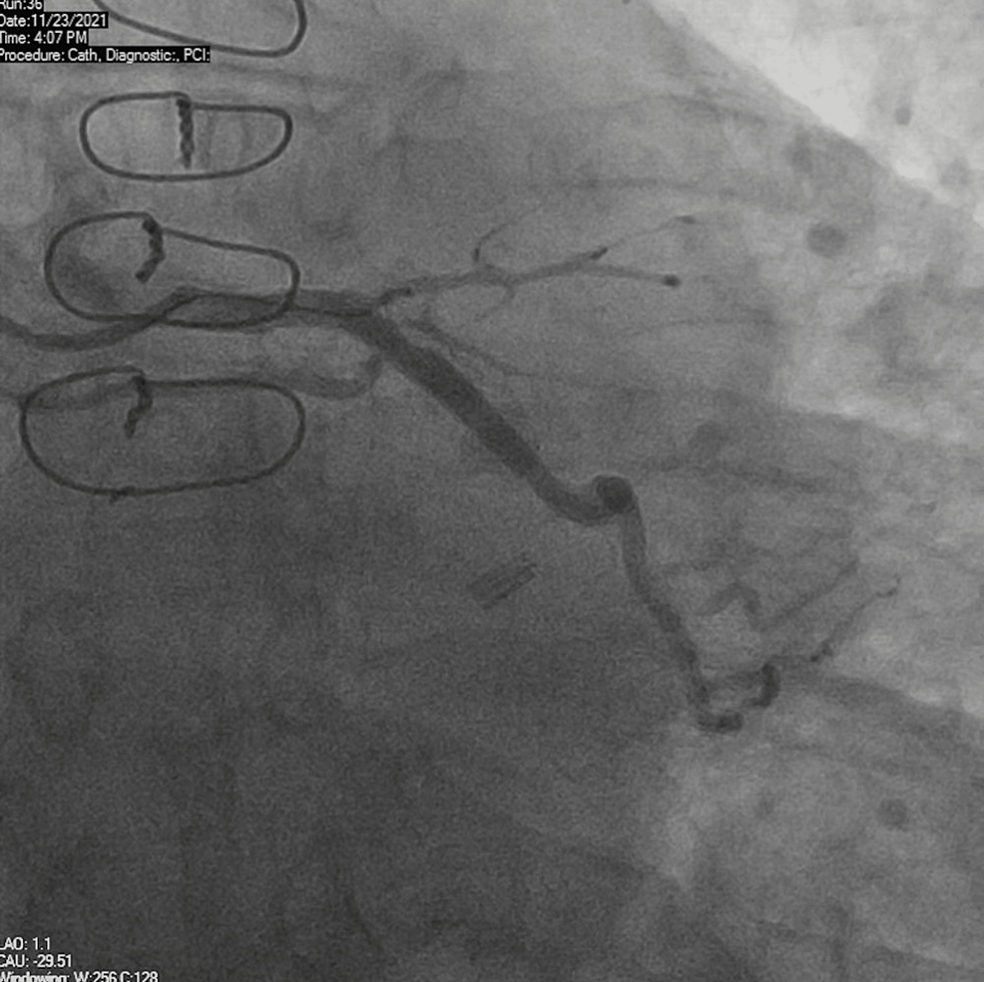
Coronary angiography showing complete sealing of the aneurysm and excellent post stent flow

The distal end of the guide extension catheter was placed distal to the area of the aneurysm with smooth delivery of the 3.5 x 26 mm PK Papyrus covered stent over it with successful sealing of the aneurysm (Figure 5).

Our patient remained asymptomatic at the three-month follow-up.

## Discussion

CAAs are usually silent, but the presentation can vary from effort angina to acute myocardial infarction from underlying atherosclerotic CAD, aneurysm thrombus formation with distal embolization, or compression of the surrounding structures [6]. Coronary angiography is the most used modality for diagnosing and managing CAAs. It provides information about the size, location, and frequency of an aneurysm and helps identify the aneurysmal thrombus and associated stenotic coronary artery lesions at the same time [7]. The management strategies available for the treatment of CAAs include medical management, percutaneous, and surgical interventions. All patients with CAAs should receive aggressive medical therapy for cardiovascular disease risk factors. Dual antiplatelet therapy should be considered if there is no contraindication. Long-term anticoagulation should also be considered if there is an intraluminal thrombus in the aneurysmal segment [6]. Smaller aneurysms (usually <10mm) are usually managed with covered stents, while surgical intervention is considered for larger aneurysms (>10mm). In our case, a large aneurysm was managed with the covered stent as the patient was deemed a poor surgical candidate by the cardiothoracic surgery team. Covered stents are usually drug-eluting stents coated on their abluminal surface from the artificial or natural material. The use of covered stents for CAAs has been challenging, and there is only small data available. We used a balloon-expandable PK Papyrus stent covered with polyurethane matrix with relatively higher bending flexibility. The risk associated with the use of covered stents includes restenosis or thrombosis, lack of flexibility, and sealing of side branches [8]. Due to the reduced deliverability and lack of flexibility with covered stents, a guide extension catheter was used to provide greater flexibility and ensure the accurate and smooth delivery of the stent. It is important to continue dual antiplatelet therapy after the placement of covered stents to prevent stent restenosis/thrombosis [9,10].

## Conclusions

CAAs are more commonly diagnosed with the advent of coronary angiography, and their management is often challenging. The management depends on the symptoms, size, and etiology of the aneurysm. The large aneurysms are usually managed surgically, while covered stenting is reserved for small aneurysms. We highlight the management of a large CAA by covered stenting in a poor surgical candidate. More studies are needed to determine the best treatment strategy for large CAAs.
